# Midterm Results of Neocuspidization of the Aortic Valve with Ozaki Technique in Adults

**DOI:** 10.21470/1678-9741-2024-0126

**Published:** 2025-10-31

**Authors:** Seguel S. Enrique, Reyes M. Rodrigo, González L. Roberto, Rubilar P. Héctor, Sepúlveda P. Camila, Barril M. Gustavo, Stockins L. Aleck

**Affiliations:** 1 Department of Surgery, Faculty of Medicine, Universidad de Concepción, Concepción, Chile; 2 Cardiovascular Center, Guillermo Grant Benavente Hospital of Concepción, Concepción, Chile; 3 Department of Medical Education, Faculty of Medicine, Universidad de Concepción, Concepción, Chile; 4 Surgery Residency Program, Pontifical Catholic University of Chile, Santiago, Chile

**Keywords:** Cardiac Surgery, Aortic Valve, Aortic Valve Repair, Aortic Valve Replacement

## Abstract

The neocuspidization technique using autologous pericardium (AVNeo^®^) is a recent alternative for aortic valve replacement in selected patients. Between 2019 and 2023, we applied it in 56 patients, evaluating surgical outcomes, survival, reintervention rates, and clinical and echocardiographic results. We analyzed its advantages, patient selection criteria, limitations, and management of bicuspid valves. We also assessed whether it is suitable for all patients and discussed the midterm outcomes observed. AVNeo^®^ may offer a promising option, especially for younger patients, by preserving native anatomy and avoiding prosthetic materials, though long-term data and further research are still needed.

## INTRODUCTION

**Table:** 

Abbreviations, Acronyms & Symbols	
CPB	= Cardiopulmonary bypass
EuroSCORE	= European System for Cardiac Operative Risk Evaluation
SD	= Standard deviation
VSD	= Ventricular septal defect

Many prosthetic models have been developed with the aim of achieving the ideal valve substitute. Among other characteristics, this substitute should have good hemodynamics (low gradient, no insufficiency), be easy to implant with a reproducible technique, not alter blood components or be thrombogenic, be durable over time, and resistant to infections.

Currently used mechanical prostheses possess some of these characteristics: they are easy to implant, have a low rate of structural deterioration, excellent hemodynamics, and long-term durability. However, they require lifelong anticoagulant treatment to prevent thrombus formation and embolisms^[1,2]^.

Tissue animal prostheses do not require anticoagulation, but they have limited durability, especially in younger patients and in situations of suboptimal hemodynamics such as small aortic roots and/or rings^[3-5]^.

Aortic homografts allow for the replacement of the diseased valve with one extracted from a cadaver, but their availability is limited, which prevents their implantation in all centers^[6]^.

The Ross procedure uses the patient's own pulmonary valve as an aortic substitute and a homograft (or another substitute) to replace the pulmonary valve. Although this technique has shown excellent long-term results, it is technically more demanding, and few centers have experience with it^[7,8]^.

The valve reconstruction procedure known as AvNeo^®^, or aortic neocuspidization, was proposed by Dr. Shigeyuki Ozaki in Japan^[9]^.

**Fig. 1:**
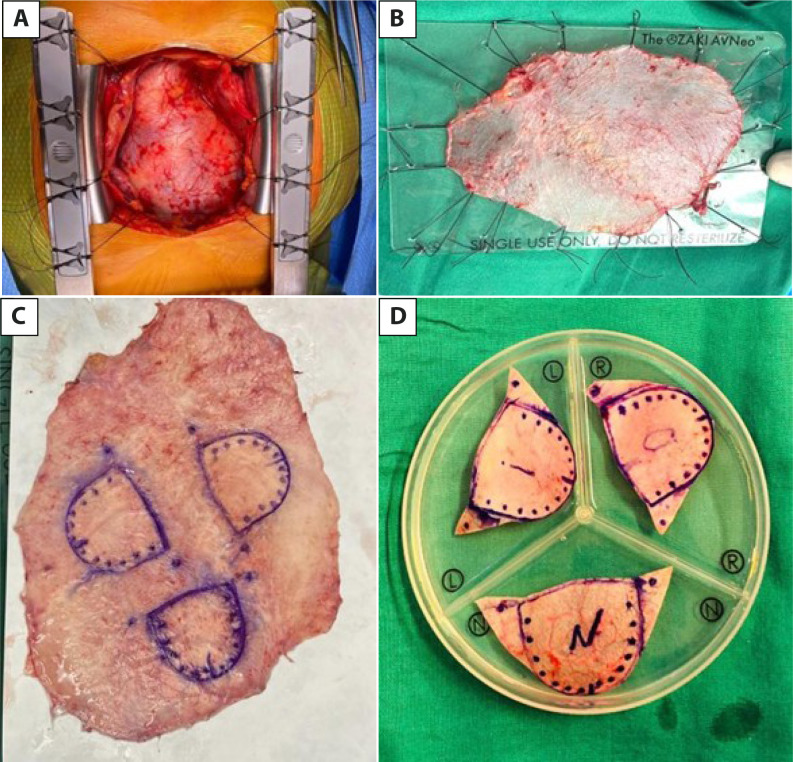
A) Exposure of the anterior pericardium. B) Stretched and fixed pericardium on the sheet for treatment with glutaraldehyde. C) Marking of the neo-cusps on the pericardium, which are subsequently cut out for use (D).

Surgery is performed under general anesthesia with standard invasive monitoring for aortic valve replacement. All patients undergo transesophageal echocardiography. The pericardium is accessed through a median sternotomy. The anterior pericardium is dissected, freeing the pleurae up to the phrenic nerves and the mediastinal fat from the diaphragm to the innominate vein. A portion of pericardium of approximately 10 × 10 cm is resected, stretched, and fixed on a medical grade acetate sheet to remove the remaining tissue (Figures 1A and B). The pericardium is treated with a 0.6% glutaraldehyde solution for 10 minutes and washed in physiological saline for six minutes three times.

During this time, the patient is connected to cardiopulmonary bypass (CPB), and the heart is protected in the usual manner. The aortic valve is accessed through an aortotomy, resected, and the annulus is decalcified.

The distance between the commissures of each cusp is measured using AvNeo^®^ system gauges (JOMDD Inc., Tokyo, Japan), designed by Dr. Ozaki. Subsequently, the cusps are drawn on the pericardium according to the measurements using the system template, and each cusp is individually trimmed (Figures 1C and D).

**Fig. 2:**
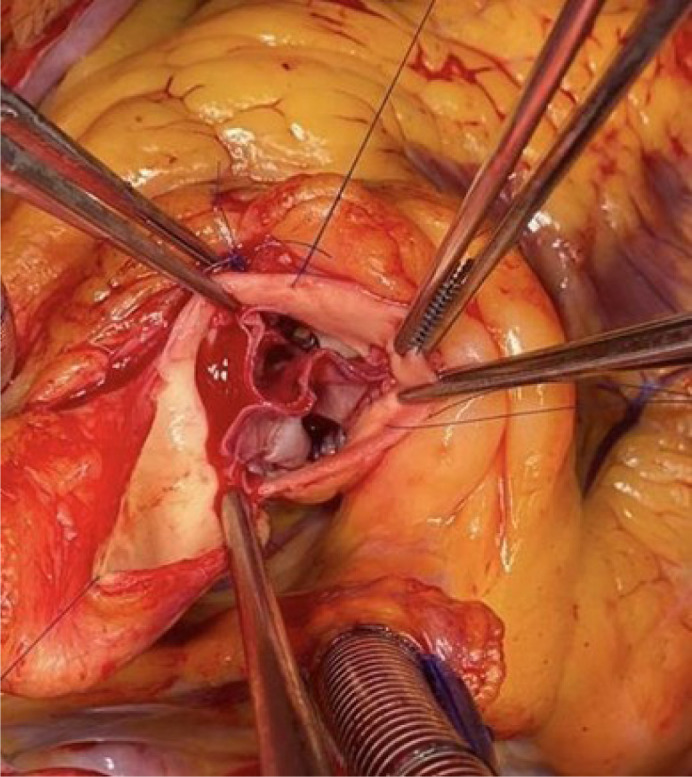
Final appearance of the valve with the three neo cusps sutured to the aortic annulus.

Each cusp is sutured to the native annulus using continuous polypropylene suture. The commissures are fixed with separate polypropylene sutures reinforced with Teflon™ pledgets that remain on the outside of the aortic wall (Figure 2).

**Fig. 3:**
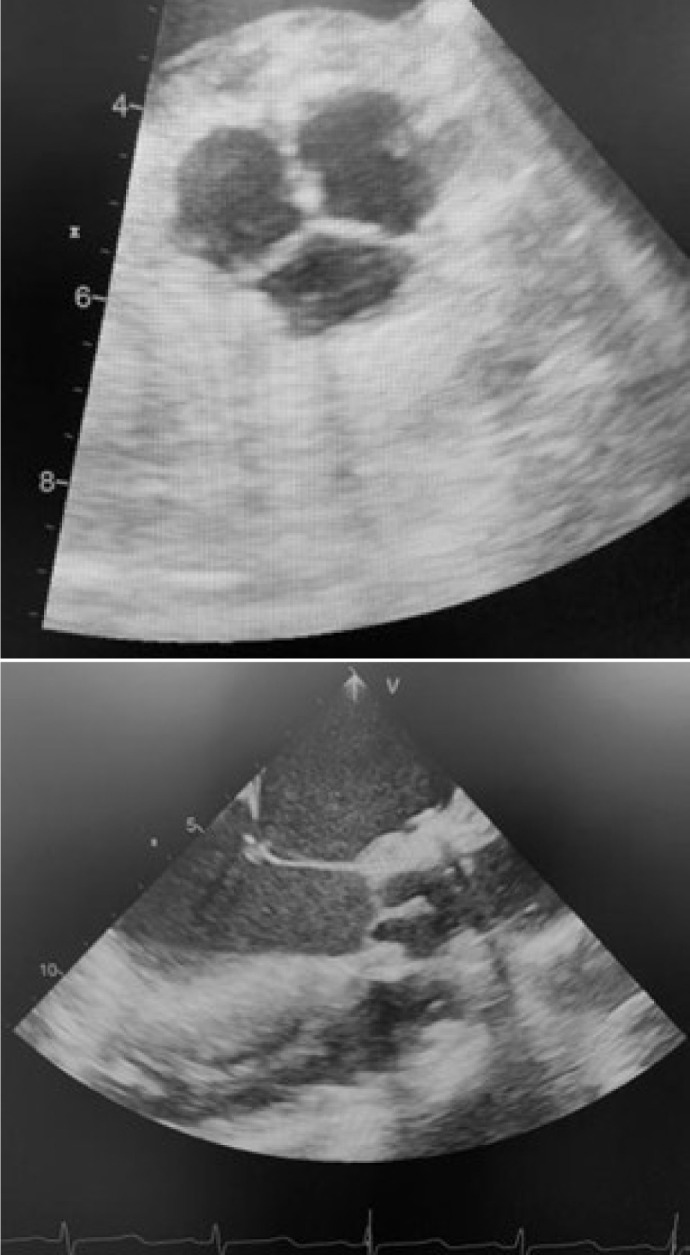
Final echocardiographic appearance in short-axis and long-axis views, showing the morphology of the valve and the wide coaptation surface.

A transesophageal echocardiogram is performed after coming off CPB to assess valve morphology, valve area, coaptation surface of the cusps, absence of insufficiency, and transvalvular gradient (Figure 3).

Technical details can be found in our previous communication^[10]^.

According to Dr. Ozaki, the technique can be performed in almost all cases, except those requiring root replacement or patients with endocarditis with ring destruction. It does not require anticoagulation, and only aspirin use (100 mg/day) for six months after surgery is recommended. Reported results show good hemodynamics, excellent survival, and a low rate of midterm reinterventions^[11]^.

### Patients

This is a descriptive study of patients who underwent aortic valve neocuspidization with the Ozaki technique at Hospital Guillermo Grant Benavente (Concepción, Chile) between March 2019 and December 2023 (n=56).

Forty-three male patients (76.8%) with a mean age of 52.6 ± 12 years (range 19 – 80) were included. Eight patients had active endocarditis, seven on native valve. The mitral valve was involved in three of them, and another was associated with a ventricular septal defect (VSD) and tricuspid endocarditis. Three patients had associated coronary artery disease, and one had a perimembranous VSD. Two patients had previous surgery (subaortic membrane and aortic valve replacement with biological prosthesis). The calculated operative risk with European System for Cardiac Operative Risk Evaluation (EuroSCORE) II was 2.3 ± 3.7%^[12]^. Excluding patients with endocarditis, the average EuroSCORE II was 1.3 ± 0.5% (Table 1).

**Table 1:** Preoperative characteristics of patients.

Sociodemographic data	Total
Sex, n (%)	
Male	43 (76.8%)
Female	13 (23.2%)
Age (x±SD)	52.6 ± 12.0
**Risk factors, n (%)**	
Hypertension	23 (41.1%)
Diabetes mellitus	7 (12.5%)
Dyslipidemia	7 (12.5%)
Smoking	5 (8.9%)
**Associated pathologies, n (%)**	
Active endocarditis	8 (14.3%)
Coronary disease	3 (5.4%)
**Previous surgery**	2 (3.8%)

SD=standard deviation

Valve pathology included stenosis in 41 and insufficiency in 15 patients. Valve morphology was bicuspid in 35 patients. For patients with aortic stenosis, maximum gradient was 81.6 ± 35 mmHg, mean gradient was 51.3 ± 18 mmHg, aortic jet velocity was 51.3 ± 18 m/s, and valve area was 0.74 ± 0.2 cm2. The average left ventricular ejection fraction was 57.6 ± 12.6% (range 27 – 68%) (Table 2).

**Table 2:** Preoperative echocardiogram.

	Total
**Valvular disease type, n (%)**	
Aortic stenosis	41 (70%)
Aortic insufficiency	15 (20%)
**Preoperative echocardiography**	
Left ventricular ejection fraction	57.6 ± 12.6%
Maximum gradient (mmHg)	81.6 ± 35
Mean gradient (mmHg)	51.3 ± 18
Aortic valve area (cm^2^)	0.74 ± 02
Aortic jet velocity (m/s)	4.53 ± 1
**Operative risk (x ± SD)**	
EuroSCORE II	2.3 ± 3.7

EuroSCORE=European System for Cardiac Operative Risk Evaluation; SD=standard deviation

The most used neo-cusp sizes were 27 mm for the left cusp, 25 mm for the right cusp, and 27 mm for the non-coronary cusp.

There were eight associated surgeries: three mitral repairs, three coronary bypasses, one tricuspid repair and closure of a perimembranous VSD, and one VSD closure.

Aortic cross-clamping and bypass times were 95.2 ± 23.7 and 102 ± 23.1 minutes, respectively. For patients without associated surgery, the times were 90.5 ± 17.2 and 97.7 ± 19.1 minutes, respectively (Table 3).

**Table 3:** Surgeries and operative results.

**Associated surgeries**	
Coronary bypass	3
Mitral valve repair	3
VSD closure + tricuspid repair	1
VSD closure	1
**Global surgical times (x ± SD)**	
Cross-clamping time (minutes)	95.2 ± 23.7
CPB time (minutes)	102 ± 23.1
**Surgical times without associated surgery**	
Cross-clamping time (minutes)	90.5 ± 17.2
CPB time (minutes)	97.7 ± 19.1
**Complications**	
Reintervention for bleeding	2
Atrial fibrillation	1
Pacemaker	0
**Operative mortality**	0

CPB=cardiopulmonary bypass; SD=standard deviation; VSD=ventricular septal defect

Postoperative echocardiography showed good valve morphology, low transaortic gradient, and absence of aortic insufficiency in all cases except three patients. One showed moderate valvular insufficiency at the commissural level. Commissural closure was performed with a suture, and subsequent control showed absence of insufficiency. Two patients had severe central insufficiency due to poor cusp coaptation, and the valve was replaced with biological prostheses in both cases.

One patient with active endocarditis was reoperated for postoperative bleeding. And one patient experienced transient atrial fibrillation.

There were no infectious, renal, neurological, or mechanical ventilation > 48 hours. There was no need for pacemakers or other cardiovascular complications.

The average length of in-hospital stay was 7.7 days, but if only patients without endocarditis are considered, the average length of stay was six days.

There was no operative mortality. Follow-up was completed until December 31, 2023. The average follow-up was 17.4 ± 12.1 months (Table 4).

**Table 4:** Follow-up.

**Follow-up**	17.4 ± 12.1 months
	(range 1 - 57 months)
**Echocardiography**	30 (53.8%) patients
Left ventricular ejection fraction	57.9 ± 13
Maximum gradient	11 ± 14.76
Mean gradient	7.4 ± 5.79
Maximum velocity	1.93 ± 0.54
Insufficiency > III	3
**Oral anticoagulation**	2
**Reintervention**	2
**Distant mortality**	1

Two patients were on anticoagulation for atrial fibrillation.

**Fig. 4:**
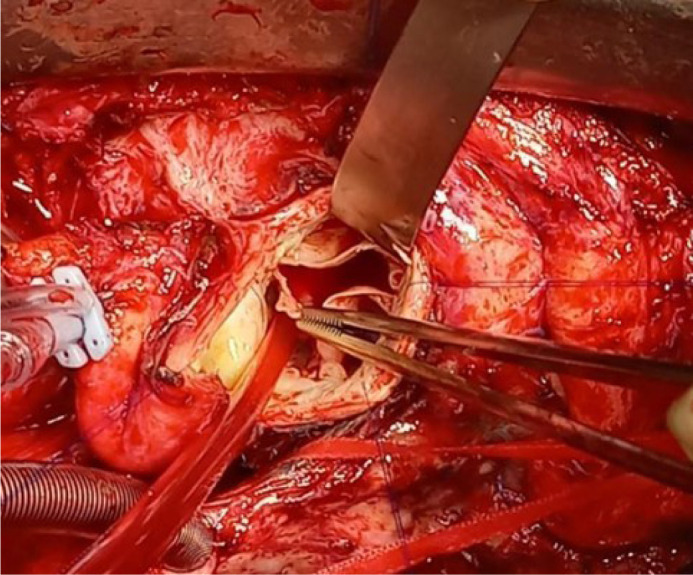
Patient reoperated due to aortic insufficiency in the postoperative period. Partial detachment of a cusp at the commissure level is observed (arrow).

**Fig. 5:**
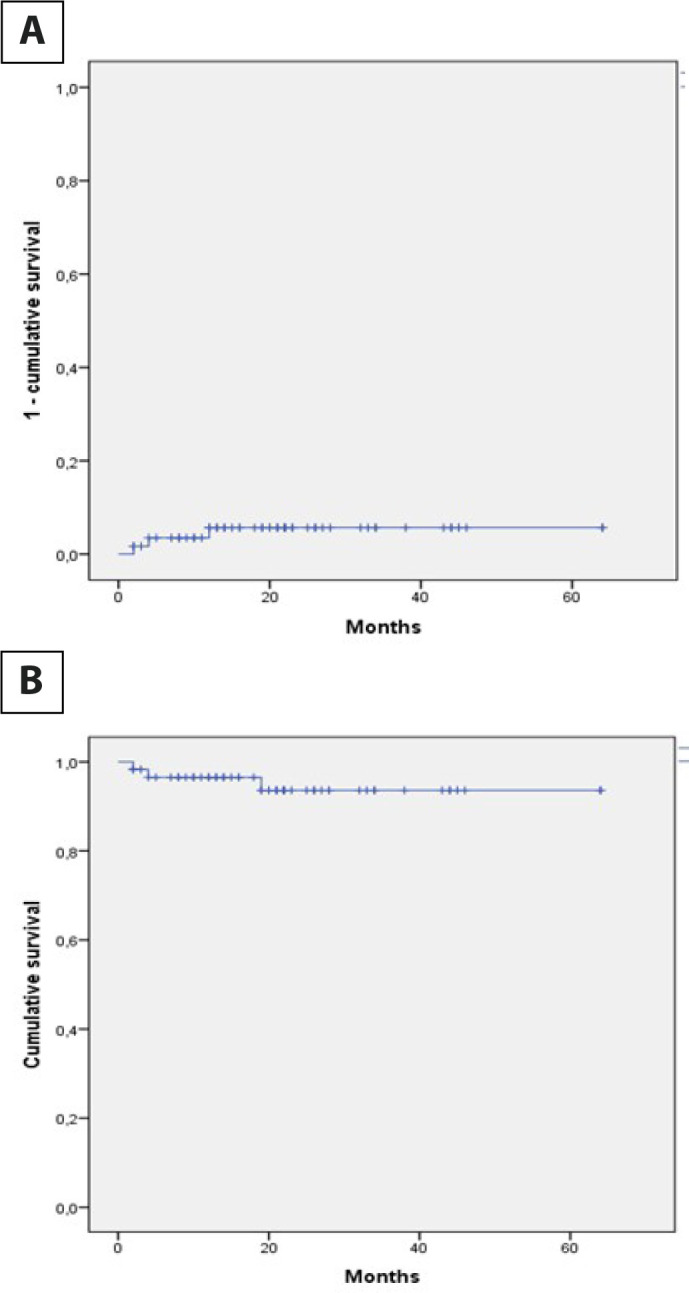
A) Curve of appearance of aortic insufficiency > 3. B) Curve of freedom from reinterventions.

There were no cases of endocarditis or cerebrovascular accidents during follow-up. Transthoracic echocardiography was performed at 12 months of follow-up in 30 patients. Valve morphology was adequate, without calcifications or deterioration of the cusps. The mean gradient was 7.4 mmHg, and the peak gradient was 11 mmHg. One patient had moderate aortic insufficiency. It was decided to follow them clinically and echocardiographically before intervening. Two patients had severe insufficiency secondary to detachment of one of the neo-cusps at the commissural level (Figure 4). Valve replacement with prostheses was performed at two and four months post-surgery. Figure 5 shows freedom from aortic insufficiency > 3 and freedom from reintervention.

Clinically, all other patients were in functional capacity I.

One patient died 52 months after surgery due to diabetic ketoacidosis. There were no deaths from cardiovascular causes during follow-up.

## QUESTIONS

- Which are the advantages of AvNeo^®^?
- Which patients can potentially benefit from this technique?
- What are the disadvantages with this technique?
- How to deal with bicuspid valves?
- Is the technique recommended for all patients?
- What were the observed midterm results?

### Discussion of Questions

**Question A.** The autologous pericardial neocuspidization proposed by Dr. Ozaki offers several advantages over current valve substitutes: it utilizes autologous pericardium, potentially reducing immune response; the cusps are sutured directly to the annulus, allowing for a larger effective orifice area; it lacks a rigid support, maintaining aortic ring mobility; and it has a wide coaptation surface, with a lower risk of insufficiency. Being biological tissue, it does not require anticoagulation^[13-15]^.

It is standardized, making the technique reproducible, and according to its author, applicable to almost all valve anatomies and pathologies^[9,10,15]^.

**Question B.** Patient selection considered those with potential benefit over standard valve replacement. Excluding the first two cases (aged 71 and 80 years), we included young or middle-aged patients (average age 51.2 years, range 19–68 years) who did not desire mechanical prosthetic replacement. In our setting, the alternative in these cases is biological prosthesis replacement, which will likely have a shorter duration than expected in patients > 65 years and will likely require reintervention in the future^[3,4,16]^.

**Question C.** The approach requires complete sternotomy for adequate pericardial dissection. This goes against the current trend of performing valve replacement surgery using minimally invasive techniques. Aortic replacement by partial sternotomy or thoracotomy has shown to decrease perioperative bleeding incidence, mechanical ventilation time, and intensive care unit stay, but has not shown an impact on reducing operative mortality in the general population^[17-19]^. These benefits are likely more significant in a higher-risk population, such as the elderly. In a low-risk population, complete sternotomy does not add additional risk and would allow for longer valve substitute durability.

**Question D.** For bicuspid valve cases, annular decalcification was performed, and a biological prosthesis sizer was used to mark the new commissures with reference to the commissure between the right and left cusps. Subsequently, the cusps were measured using these marks as a reference. This allows for the implantation of three neo-cusps of similar sizes, achieving a more symmetric valve anatomy. Attention should be paid to the fact that in these patients, the non-coronary cusp annulus usually has a deeper nadir than the others. To avoid distortion in the final height of the neo-cusps (as occurred in one case), this cusp should be sutured to the aortic wall approximately at the level of the nadirs of the right and left cusps.

**Question E.** Because the technique requires separate implantation of each cusp and construction of the commissures, aortic cross-clamping and CPB times are longer than those of a routine valve replacement. This should be considered when selecting the patient and not including patients with ventricular dysfunction, where prolonged ischemic time could result in myocardial damage and difficulty in weaning from bypass.

Two cases had severe postoperative insufficiency. In both cases, the native valves were tricuspid, with annular dilation. Likely, the neo-cusp measurements were inadequate, and smaller sizes were selected than required for those ring sizes, resulting in lack of cusp coaptation. To avoid prolonging cross-clamping and CPB times, replacement with biological prostheses was decided in both cases.

The rate of complications and operative mortality was low, and the hospital stay for elective patients was short, as expected for a series of selected, low-risk patients (excluding patients with endocarditis, the average EuroSCORE II was 1.3%).

Question F. The follow-up for this series is still brief. The clinical evolution of the patients has been very good. Echocardiograms have shown excellent valve morphology, with good cusp mobility and low transvalvular gradients.

Mylonakis et al.^[20]^, in a meta-analysis published in 2023 including 1,891 adult and pediatric patients, observed that the average effective orifice area was 2.08 ± 0.5 cm^2^/m^2^, and the maximum gradient was 15.7 ± 7.4 mmHg. The rate of moderate insufficiency observed was 0.25%.

Three cases presented aortic insufficiency on postoperative follow-up: one patient was admitted for decompensated heart failure two months after surgery, was medically compensated, and underwent an echocardiogram showing severe aortic insufficiency due to cusp prolapse. A second case consulted at four months after surgery for dyspnea. Echocardiography also showed severe insufficiency, and reoperation was decided. In the reoperation of both cases, partial detachment (5 mm) of the non-coronary cusp at a commissural level was confirmed. Valve replacement with biological prostheses was decided in the first case and mechanical in the second. However, it is likely that the cusp could have been repaired by a surgeon with more experience with the technique.

In the third case, a murmur was auscultated on clinical follow-up. Echocardiography showed moderate to severe aortic insufficiency due to non-coronary cusp coaptation deficiency. This patient is asymptomatic, there has been no ventricular dilatation, and systolic function is normal. Clinical and echocardiographic follow-up was decided.

At the time of follow-up, there were no deaths from cardiovascular causes in our series. This is likely due to the selection of young patients for the technique.

In Mylonakis' meta-analysis, with an average follow-up of 38.1 ± 23.8 months, mortality was 1.91%, and freedom from reintervention survival was 96.7%.

In Dr. Ozaki's series, which included 850 patients (average age 71 years, average follow-up of 53.7 months), actuarial survival was 85.9%, the reintervention rate was 4.2%, and the incidence of moderate to severe valvular insufficiency was 7.3% at 10 years.

In a study comparing 627 patients from Dr. Ozaki's series with 627 matched patients from the Cleveland Clinic's Perimount^®^ aortic valve replacement registry, it was observed that patients undergoing the technique had lower gradients (17 mmHg *vs.* 28 mmHg, *P*<0.001), a higher rate of insufficiency (3.6% *vs.* 1%, *P*=0.006), with similar reintervention-free survival at six years of follow-up^[21]^.

## BRIEF CONSIDERATIONS OF THE CASES REPORTED

This study involves a group of selected patients with low operative risk and good ventricular function, which limits the generalization of the findings to higher-risk populations or those with ventricular dysfunction. Additionally, the experience of the surgical team may influence the results, potentially not reflecting the reality in other centers with less or more experience in the technique of aortic valve neocuspidization.

Since aortic valve disease is a chronic condition that may require long-term monitoring, it is crucial to evaluate medium- and long-term outcomes to fully understand the effectiveness and durability of this surgical technique.

Despite these limitations, the immediate and midterm results of aortic valve neocuspidization surgery are encouraging. The technique proves to be reproducible and offers good outcomes in selected patients, especially those who wish to avoid the use of anticoagulants, such as young patients.

Further studies with a larger number of patients, longer-term follow-up, and in more diverse populations are needed to confirm these findings and the theoretical advantages of the technique over biological prostheses (durability, hemodynamic behavior, reinterventions) and to establish the definitive role of this technique in the treatment of aortic valve disease.

## LEARNING POINTS

- AVNeo^®^ may offer a promising option, by preserving native anatomy, avoiding prosthetic materials and anticoagulation.
- Long-term data and further research are still needed to determine which patients can potentially benefit from this technique.

## Data Availability

The authors declare that the data will be available upon request to the authors.
